# Preservation of Ceriporiopsis subvermispora and Lentinula edodes treated wheat straw under anaerobic conditions

**DOI:** 10.1002/jsfa.8745

**Published:** 2017-11-22

**Authors:** Lei Mao, Anton S M Sonnenberg, Wouter H Hendriks, John W Cone

**Affiliations:** ^1^ Animal Nutrition Group Wageningen University & Research Wageningen The Netherlands; ^2^ Plant Breeding Wageningen University & Research Wageningen The Netherlands

**Keywords:** Ceriporiopsis subvermispora, Lentinula edodes, wheat straw, in vitro gas production, anaerobic storage

## Abstract

**BACKGROUND:**

No attention has been paid so far to the preservation of fungal‐treated lignocellulose for longer periods. In the present study, we treated wheat straw (WS) with the white‐rot fungi Ceriporiopsis subvermispora and Lentinula edodes for 8 weeks and assessed changes in pH, chemical composition and in vitro gas production (IVGP) weekly. Fungal‐treated WS was also stored for 64 days ‘as is’, with the addition of lactic acid bacteria (LAB) or with a combination of LAB and molasses in airtight glass jars mimicking ensiling conditions.

**RESULTS:**

Both fungi significantly reduced the lignin and hemicellulose content of WS, and increased the cellulose content. The IVGP increased with increasing time of incubation, indicating the increase in digestibility. Both fungi lowered the pH of WS under 4.3, which guarantees an initial and stable low pH during anaerobic storage. Minor changes in fibre composition and IVGP were observed for stored L. edodes treated WS, whereas no change occurred for C. subvermispora.

**CONCLUSION:**

It is possible to conserve C. subvermispora and L. edodes treated straw under anaerobic condition without additives up to 64 days. This finding is important for practical application to supply fungi‐treated feed to ruminant animals for a prolonged period. © 2017 The Authors. *Journal of The Science of Food and Agriculture* published by John Wiley & Sons Ltd on behalf of Society of Chemical Industry.

## INTRODUCTION

Large quantities of agricultural by‐products (e.g. rice and wheat straws) are produced every year and most of it is left on the field or burned. However, both rice and wheat straws (WS) can be potential valuable feedstuffs for ruminants, as the major part consists of cellulose and hemicellulose.^1^, ^2^ These carbohydrates are to a limited extent used by rumen microorganisms because cereal straw contains high levels of lignin with complex linkages to the carbohydrates.^3^ Lignin itself cannot be degraded under anaerobic conditions in the rumen and its concentration is negatively correlated with cell wall degradability.^4^ Therefore, different methods have been used to make the carbohydrates more accessible to rumen microbiota, including physical, chemical, physicochemical and biological methods.^2^, ^5^ A promising low tech and low cost method for upgrading low value, high lignocellulose‐containing biomass is the selective lignin degradation by white‐rot fungi.^6^–^8^ White‐rot fungi are the only organisms that are able to degrade lignin effectively by producing extracellular enzymes, such as lignin peroxidase, manganese peroxidase and laccase.^9^ Studies found that the nutritive value and degradability of cereal straw improved after a fungal treatment.^6^, ^7^, ^10^ Fungi can degrade lignin aerobically during an incubation period of several weeks depending on the species and fungal strain. After successful lignin degradation, termination of fungal activity is essential to maintain the nutritive value for ruminants. In addition, to make fungal‐treated biomass available as a feed ingredient for ruminants over a prolonged period of time, successful conservation is essential.

Ensiling is widely used as a method to preserve forages, such as grass and maize for ruminants. The process of ensiling is based on the anaerobic fermentation of sugars by lactic acid bacteria (LAB), which produce lactic acid and decrease the pH to around 4. The acidic environment effectively inhibits the proliferation and fermentation of other undesirable microorganisms, such as yeasts, enterobacteria and clostridia. Forages can be well preserved with minimum nutritive losses during ensiling^11^ and the palatability increases by the formation of lactic acid. The number of lactic acid bacteria and amount of soluble sugars are important to achieve a fast decrease in pH in the silage. However, fermentation might be hampered for substrates with a low content of epiphytic LAB and sugar. In those cases, LAB and molasses (sugars) can be added to the silages.^12^, ^13^

The present study aimed to determine the possibility to store fungal‐treated WS under anaerobic conditions ‘as is’, with the addition of LAB or with a combination of LAB and molasses.

## MATERIALS AND METHODS

### Fungal strains and spawn preparation

The fungi *Ceriporiopsis subvermispora* (strain code: CBS 347.63; Origin: USA) and *Lentinula edodes* (strain code: CCBAS389; Origin: Czech Republic) were selected for the present study because they have been shown to have a greater ability to degrade lignin in lignified biomass (including WS) compared to other investigated white‐rot fungi.^6^ The spawn was prepared as described previously by Van Kuijk *et al*.^10^

### Fungal solid state fermentation and storage study

Conventional WS was used as substrate for the solid state fermentation with the two fungi. The straw was chopped to a length of approximate 0.5 cm and submerged in water for 3 days, after which the water was drained over a 5‐h period. The wet WS was mixed, distributed into plastic containers (3000 mL, with cover: 195 × 195 mm, base: 185 × 185 mm, height: 112 mm; model TP3000 + TPD3000; Combiness, Nazareth, Belgium) containing a filter and autoclaved at 121 °C for 1 h. After cooling to room temperature, 12–13 g (10% of dry WS) of spawn was added to each box and mixed gently by hand under aseptic conditions. In the first experiment, each container was filled with approximately 544 g of wet WS [dry matter (DM) content of autoclaved WS (AWS) was 190.7 g kg^–1^]. The containers were incubated in a climate controlled room (24 °C) for 8 weeks, and three containers inoculated with each fungus were collected each week to determine pH, chemical composition and *in vitro* gas production (IVGP).

In the second experiment, each container was filled with approximately 541 g (*C. subvermispora*) and 531 g (*L. edodes*) wet WS of the same batch as Experiment 1. The DM content of the AWS was 180.2 g kg^–1^. Wheat straw was incubated with *C. subvermispora* and *L. edodes* for 39 and 52 days, respectively. Untreated WS, AWS (121 °C for 1 h) and AWS treated with *C. subvermispora* and *L. edodes* were then packed into 500‐mL airtight glass jars ‘as is’, with 1 × 10^6^ colony‐forming units *Lactobacillus plantarum* g^–1^ wet substrate or with a combination of LAB and molasses (3 % wet weight). The jars were filled by pressing the substrate into the jars, leaving as little air as possible before being closed and stored at 20 °C in a climate‐controlled chamber. Three jars of each treatment were collected at 0, 2, 4, 8, 16, 32 and 64 days of storage for analysis with pH being determined directly in all collected samples as described below. Chemical analysis was conducted on air dried samples while volatile fatty acids (VFA) and ammonia (NH_3_‐N) were determined on fresh samples from day 0 and 64.

### Analytical methods

Samples were air dried at 70 °C until constant weight and ground in a hammer mill over a 1 mm sieve (Peppink 100 AN; Peppink Mills BV, Olst, The Netherlands). The DM content was determined after drying at 103 °C for 4 h (ISO 6496, 1999)^14^ and ash by incineration at 550 °C for 3 h (ISO 5984, 2002).^15^ Neutral detergent fiber (NDF), acid detergent fiber (ADF) and acid detergent lignin (ADL) were determined by the methods of Van Soest *et al*.,^16^ using an Ankom fiber analyzer (ANKOM 2000 I fibre analyzer; ANKOM Technology, Macedon, NY, USA). In short, NDF was determined by boiling the dried material with ND‐reagent with the addition of a heat stable amylase. The insoluble residue was designated as the NDF fraction. ADF comprised the insoluble fraction after boiling the material in AD‐reagent. The ADF fraction was subsequently incubated with 72% sulphuric acid for 3 h at 20 °C and the insoluble fraction was designated as ADL. Each fibre fraction was corrected for ash content. Cellulose was calculated as the difference between ADF and ADL and hemicellulose as the difference between NDF and ADF. Nitrogen content was determined using the Kjeldahl method with CuSO_4_ as catalyst and crude protein (CP) was calculated as N × 6.25 (ISO 5983, 2005).^17^

Next, 30 g of fresh straw was weighed into a stomacher bag and 270 mL of demineralised (demi) water was added, followed by mixing in a stomacher (400 Circulator; Seward, Worthing, UK) at 230 r.p.m. for 5 min after which the pH was measured (Model HI 9024; Hanna Instruments, IJsselstein, The Netherlands). In the second experiment, the samples were treated in the same manner, 0.6 mL of liquid was collected and mixed vigorously with an equal volume (1:1, v/v) of trichloroacetic acid (10%) for analysis of NH_3_‐N, or for analysis of VFA mixed with an internal standard solution (85% ortho‐phosphoric acid containing 19.681 mmol L^–1^ isocapronic acid). The mixtures were stored at –20 °C until analysis.

For NH_3_‐N analysis, the frozen samples were thawed, followed by centrifugation at 14 000 × *g* for 10 min. The colorimetric method, described by Scheiner^18^ was used to determine NH_3_‐N at 623 nm using a spectrophotometer (Evaluation 201; Thermo Fisher Scientific, Waltham, USA). To determine VFA concentration, the thawed samples were centrifuged at 14 000 × *g* for 5 min and the supernatant was used to measure the concentration of VFA by gas chromatography (Trace GC; Interscience, Milan, Italy) with detection by a flame ionization, as described by Pellikaan *et al*.,^19^ using hydrogen as the carrier gas instead of helium.

The amount of base used to increase the pH to neutral (pH 7) was determined in 30 mL of the stomacher solution. A Titrando machine (in conjunction with tiamo software) consist of 907 Titrando, 800 Dosino and 801 Stirrer (Metrohm AG, Herisau, Swizerland) was used to determine the amount of NaOH required to increase the pH to 7 by titrating with 0.1 mol L^–1^ NaOH (Titrisol sodium hydroxide solution; Merck, Darmstadt, Germany). Data were expressed as mmol NaOH required to change the initial pH to 7 per kg of DM of the original stored sample.

### In vitro gas production

*In vitro* gas production was performed as described by Cone *et al*.^20^ In brief, rumen fluid was collected from three lactating, rumen fistulated cows fed *ad libitum* corn silage and grass silage. The strained rumen fluid was filtered through two layers of cheese cloth and mixed with a mineral buffer solution. All procedures were conducted under continuous flushing with CO_2_. Samples were incubated with the buffered rumen fluid for 72 h and gas production was automatically recorded. Gas production was corrected for blank gas production (i.e. gas production in buffered rumen fluid without sample) to allow for fermentation of residual organic matter (OM) in the rumen fluid.

### Statistical analysis

In Experiment 1, chemical composition and gas production of each fungal treatment were subjected to a general linear model in SAS, version 9.3 (SAS Institute Inc., Cary, NC, USA):

$$Y_{\mathrm{ij}}=µ+\alpha_{i}+\omega_{\mathrm{ij}}$$

where *Y*_ij_ is observation *j* in treatment *i*, *μ* is the overall mean, *α*_i_ is the fix effect of time and *ω*_ij_ is the random error. Multiple comparisons using Tukey's significant test with *α* = 0.05 in the LSMEANS statement were used to determine significance between treatments.

In Experiment 2, independent sample *t*‐tests in SAS, version 9.3, were used to compare the difference between 0 and 64 days of storage.

## RESULTS AND DISCUSSION

### Fungal fermentation of wheat straw

#### pH change of wheat straw after fungal treatment

**Figure 1:**
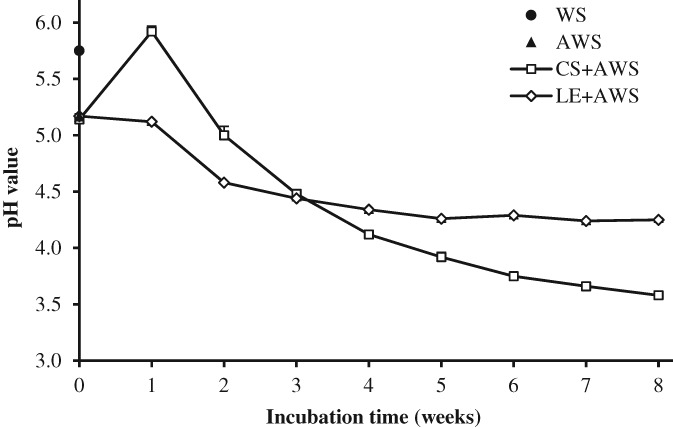
pH value of wheat straw (WS), autoclaved wheat straw (AWS), C. subvermispora treated AWS (CS + AWS) and L. edodes treated AWS (LE + AWS) for 0–8 weeks (week 0 represents AWS inoculated with spawn without incubation). Error bars indicate the SD.

After autoclaving, the pH of the WS decreased from 5.75 to 5.17 (Fig. 1). The pH of the AWS treated with *C. subvermispora* showed an increase from week 0 (5.14) to 1 (5.92), and then gradually decreased to 3.58 after 8 weeks of incubation. The *L. edodes* treated straw showed a slight decrease in pH during the first week, followed by a more pronounced decrease to 4.26 until 5 weeks of incubation, after which the pH remained relatively stable. Apparently, both fungi produced organic acids during the solid state fermentation. Zadražil^21^ reported a decrease in pH of WS after treatment with the basidiomycetes *Pleurotus cornucopiae, Pleurotus* sp. Florida, *Agrocybe aegerita* and *Stropharia rugoso‐annulata*. In that study, a slight initial increase in pH was also observed, which then remained stable or was decreased by some fungi. A decrease in pH by the fungi *Dichomitus squalens*, *Trametes ochracea* and *Trametes versicolor* on wood chips was also reported by Mäkelä *et al*.^22^ Although no data on pH change in WS treated with *C. subvermispora* have been reported, many studies have shown that *C. subvermispora* produces organic acids during growth and colonization of a substrate.^22^–^24^ Particular acids involved in lignin degradation produced by this fungus are ceriporic acids, which are acids with an itaconic core and different lengths of the alkyl side chains.^25^, ^26^ Hermann *et al*.^27^ reported a similar pH change for the growth of *L. edodes* on sawdust. Although no in depth analyses of the acid production have been conducted for *L. edodes* as far as we know, this fungus is known to produce oxalic acids.^28^

Acids produced by fungi have diverse functions. Apart from their role in anabolic processes and cellular physiology,^29^ acids are excreted to lower the environmental pH to inhibit the growth of competitors allowing fungi to dominate rapidly as the major microorganism.^30^ In addition, some organic acids, such as oxalic acid, also have a vital role in lignin degradation.^22^, ^24^, ^31^ The lowering of pH by fungi during colonization of WS might also have a preservative effect, which might be beneficial for the storage of treated material for use as a feed ingredient.

#### Chemical composition

The changes in the chemical composition during solid state fermentation with *C. subvermispora* and *L. edodes* are shown in Tables 1 and 2. The difference in the chemical composition at week 0 of two inoculated fungi was most likely a result of variation in the WS that we used for each fungal inoculation, even though the straw was collected from the same batch. The hemicellulose and lignin content decreased during the 8 weeks of incubation for both fungi. As a consequence, a significant increase in cellulose, CP and ash content was observed. The increase in ash content indicates the loss of OM during the fungal treatment. Fungi convert 40–50% of the carbon in carbohydrates into carbon dioxide and this is likely the main cause of OM losses.^32^ The increase in CP is thus likely not an absolute increase but an enrichment as a result of the loss of carbon. This was indicated by the results of a study by Van Kuijk *et al*.^10^ showing that the CP concentration significantly increased in *L. edodes* treated regular WS from 0 to 12 weeks of incubation, whereas the absolute amount of CP did not change. Similar results have been obtained by our research group for both *C. subvermispora* and *L. edodes* treated organic WS (Mao L *et al*., unpublished). In addition, CP will be an overestimation of protein because part of the nitrogen will be used to generate mycelial biomass. A substantial part of N is incorporated in chitin in the fungal cell wall and thus not as protein. Changes in the chemical composition of WS after treatment with *C. subvermispora* or *L. edodes* have already been reported^6^, ^10^ and are in line with the present study.

**Table 1:** Chemical composition of C. subvermispora treated autoclaved wheat straw from 0 to 8 weeks

Time (weeks)	Chemical composition				
	Ash	Crude protein	Cellulose	Hemicellulose	Lignin
0	38.8 d	27.8 d	467.0 c	298.5 a	76.9 ab
1	38.8 d	27.3 d	473.6 bc	296.9 a	81.4 a
2	38.8 d	29.1 cd	465.4 c	295.0 a	79.4 ab
3	39.7 d	31.0 bcd	466.4 c	270.5 b	74.0 b
4	40.7 c	31.9 abc	466.6 c	248.3 c	59.6 c
5	40.8 c	32.6 abc	472.0 bc	224.2 d	45.7 d
6	42.4 b	33.5 ab	471.6 bc	200.3 e	36.0 e
7	42.7 b	34.4 ab	484.7 ab	166.7 f	26.8 f
8	43.9 a	35.0 a	494.2 a	145.9 g	22.0 f
RMSE	0.34	1.32	5.02	4.43	2.28
*P*	< 0.0001	< 0.0001	< 0.0001	< 0.0001	< 0.0001

Ash, g kg^–1^ dry matter; crude protein, cellulose, hemicellulose and lignin, g kg^–1^ organic matter.

RMSE, root mean square error.

Values within a column with different lowercase letters are significantly different (*P* < 0.05).

**Table 2:** Chemical composition of L. edodes treated autoclaved wheat straw from 0 to 8 weeks

Time (weeks)	Chemical composition				
	Ash	Crude protein	Cellulose	Hemicellulose	Lignin
0	54.0 d	32.8 d	470.6 c	299.4 a	79.1 ab
1	56.1 cd	34.3 cd	470.7 c	293.9 ab	82.4 a
2	58.4 c	36.0 bc	485.7 ab	279.7 bc	83.6 a
3	61.2 b	36.0 bc	489.8 ab	268.3 cd	81.1 ab
4	61.7 ab	36.1 bc	484.9 b	262.2 d	74.1 b
5	62.3 ab	38.4 ab	494.5 ab	240.7 e	59.3 c
6	63.8 a	39.1 a	488.9 ab	235.2 ef	57.1 cd
7	63.7 a	37.9 ab	498.3 a	220.2 fg	49.2 de
8	64.1 a	38.9 a	496.6 ab	215.5 g	48.6 e
RMSE	0.89	0.95	4.40	5.71	2.79
*P*	< 0.0001	< 0.0001	< 0.0001	< 0.0001	< 0.0001

Ash, g kg^–1^ dry matter; crude protein, cellulose, hemicellulose and lignin, g kg^–1^ organic matter.

RMSE, root mean square error.

Values within a column with different lowercase letters are significantly different (*P* < 0.05).

#### In vitro gas production

**Figure 2:**
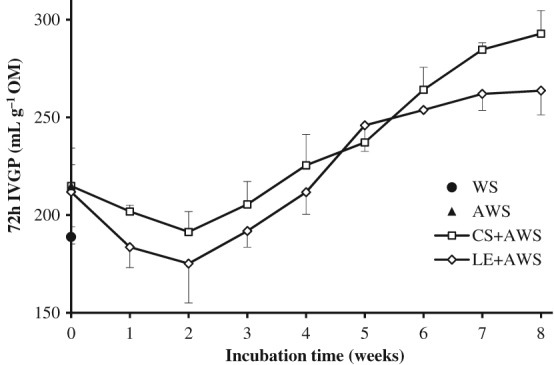
Total gas production after 72 h of wheat straw (WS), autoclaved wheat straw (AWS), C. subvermispora treated AWS (CS + AWS) and L. edodes treated AWS (LE + AWS) for 0–8 weeks (week 0 represents AWS inoculated with spawn without incubation). Error bars represent the SD.

The IVGP simulates fermentation in the rumen because there is a linear relationship between gas production and OM degradation.^20^ The total gas production after 72 h of incubation caused by fermentation of WS treated with *C. subvermispora* and *L. edodes* for different weeks is shown in Fig. 2. The 72‐h gas production with untreated WS and AWS was 188.8 and 214.9 mL g^–1^ OM, respectively. Because autoclaving of WS mimics a thermal treatment, it will increase the enzymatic digestibility^33^ and thus might have a positive effect on the IVGP. The gas production of the AWS treated with *C. subvermispora* increased to 292.8 mL g^–1^ OM and that treated with with *L. edodes* increased to 263.7 mL g^–1^ OM at 8 weeks of incubation. The gas production of treated WS decreased until 2 weeks and increased thereafter. The increased gas production after 8 weeks of fungal treatment shows that the nutritive value of the WS increased as a consequence of the decreased lignin content. The decrease in IVGP in the first 2 weeks of fungal treatment indicates a decrease in easily accessible carbohydrates likely consumed first by the fungi before lignin is degraded. Comparable results were reported by Tuyen *et al*.^6^ and Van Kuijk *et al*.,^10^ who showed that gas production of WS increased by treatment of the straw with *C. subvermispora* and *L. edodes* and with a drop in gas production after only 1 or 2 weeks of fungal incubation.

### Fungal‐treated wheat straw stored under anaerobic conditions

#### pH change during storage

**Figure 3:**
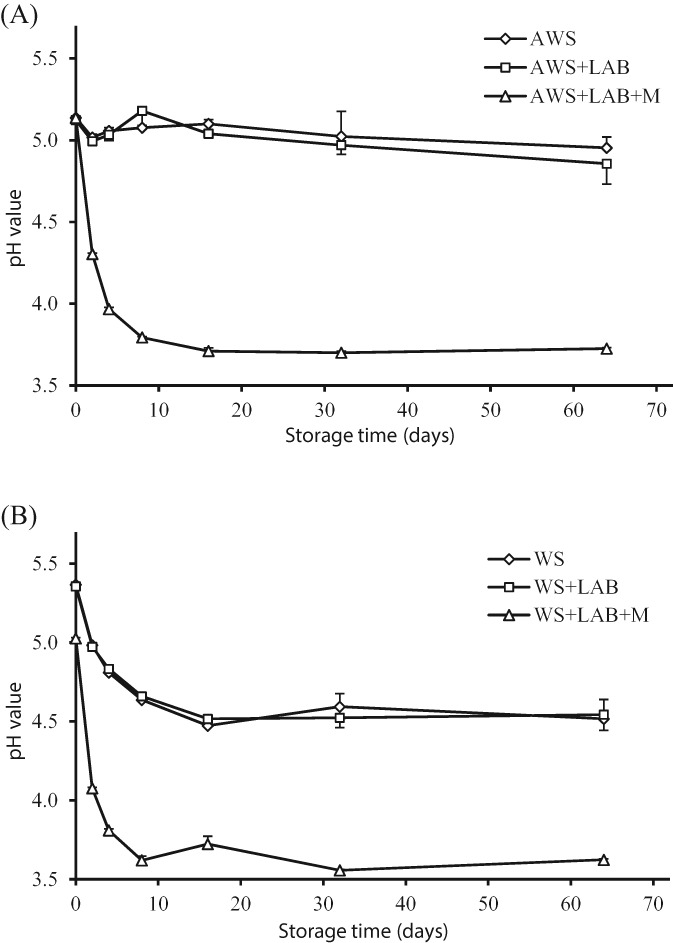
pH value of (A) untreated autoclaved wheat straw (AWS) and (B) wheat straw (WS) after 0, 2, 4, 8, 16, 32 and 64 days of storage. Substrates were stored ‘as is’, with the addition of lactic acid bacteria (LAB) or with the addition of a combination of LAB and molasses (LAB + M). Error bars represent the SD.

**Figure 4:**
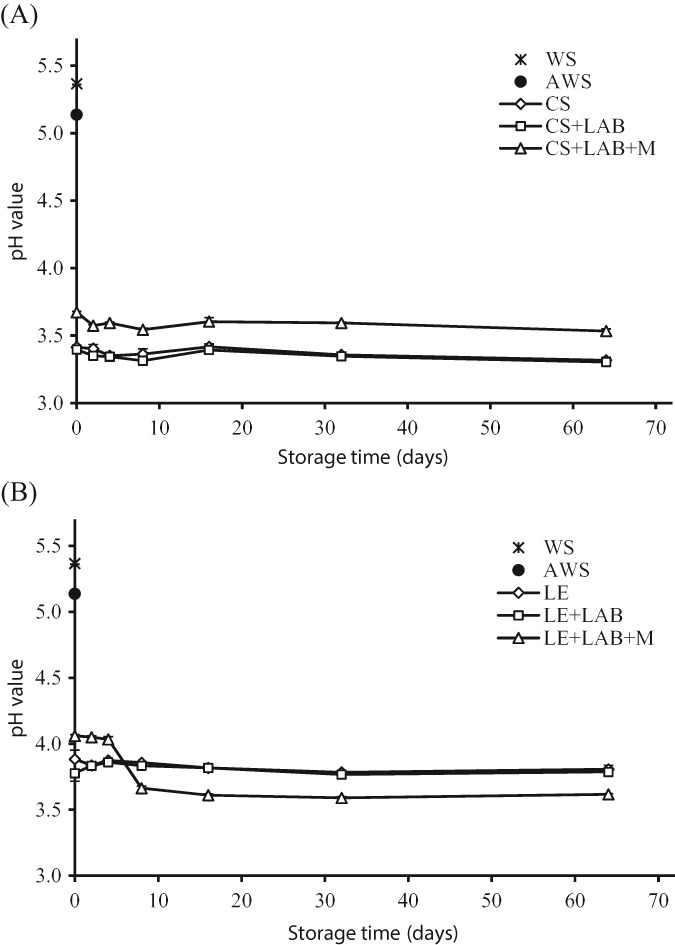
pH value of autoclaved wheat straw treated with (A) C. subvermispora (CS) and (B) L. edodes (LE) after 0, 2, 4, 8, 16, 32 and 64 days of storage. Substrates were stored ‘as is’, with the addition of lactic acid bacteria (LAB) or with the addition of a combination of LAB and molasses (LAB + M). Treatment of AWS and WS are described in Fig. 3. Error bars represent the SD.

To determine whether the fungal treatment has a preservative effect, different parameters were monitored during anaerobic storage of the fungal‐treated WS. The changes in pH of the untreated and treated WS during the storage process are shown in Figs 3 and 4. The pH of the AWS ‘as is’, with LAB (AWS + LAB) and with a combination of LAB and molasses (AWS + LAB + M) at day 0 of the storage process was 5.14, 5.12 and 5.14, respectively (Fig. 3A). After 64 days, the pH of AWS and AWS + LAB decreased slightly to 4.95 and 4.86, respectively. By contrast, the pH of AWS + LAB + M showed a rapid decrease to 3.79 on day 8, followed by a period with a stable pH (∼3.7) up to 64 days. For the non‐AWS, the pH showed a similar pattern during the anaerobic storage as the AWS treatment (Fig. 3B), except that, for all treatments, a lower pH was reached. This might be because sterilisation extracts some easily accessible carbohydrates that can be used by the LAB to generate lactic acids. It is unclear, however, why the pH decreases also without the addition of LAB. It is obvious that untreated WS does not contain many nutrients for LAB and that molasses are needed to generate a large pH drop for a stable storage.

The solid state fermentation of straw for 39 days with *C. subvermispora* in Experiment 2 resulted in a lower pH (3.41) compared to the pH (3.58 at 8 weeks) in Experiment 1 and the pH remained stable during the storage process. The addition of LAB and LAB + M hardly influenced the pH (Fig. 4A). A similar trend was observed for *L. edodes* (Fig. 4B), with a pH of 3.88 after 52 days of aerobic solid state fermentation. A higher value (4.25) was showed in Experiment 1 after 8 weeks of incubation. The addition of LAB + M to *L. edodes* treated AWS resulted in a small decrease in pH, from 4.06 to 3.66 after 8 days of storage, reaching the same value as the *C. subvermispora* treated WS, indicating that this pH is reached by LAB using only the molasses and thus independent of the type of fungal‐treated WS. These results show that the decrease in pH by both fungi is sufficient to conserve fungal‐treated WS under anaerobic conditions for a prolonged period without the need for additives. Studies on the combination of solid state fermentation and ensiling process are rare. Yang *et al*.^34^ used *Penicillium decumbens* as inoculate with a 9:1 ratio of corn straw and wheat bran. After solid state fermentation, the pH reached of 6.3 and addition of LAB and molasses was required to reach a pH drop to 4.5 within 1 day. Another example of a combination of an ensiling process and a fungal treatment was published by Thomsen *et al*.,^35^ who ensiled WS for 4 weeks by adding *L. buchneri* and xylose as a carbon source. The ensiling had a positive effect on the enzymatic degradation of WS and on ethanol production. However, a significant positive effect was only reached if the ensiled material was washed with water before fungal inoculation.

#### Chemical composition and gas production of fungal‐treated wheat straw during storage

The overall chemical composition of untreated and fungal‐treated WS did not change much during the 64 days of anaerobic preservation (Tables 3–6). Significant, but small changes were observed in some cases for lignin, cellulose and hemicellulose. However, these changes in chemical composition were not systematic.

**Table 3:** Chemical composition, in vitro gas production (IVGP) and silage characteristics of wheat straw (WS) stored anaerobically ‘as is’, with the addition of lactic acid bacteria (LAB) or with the addition of a combination of LAB and molasses (LAB + M) for 0 (T0) and 64 (T64) days

Treatment	WS ‘as is’		WS + LAB		WS + LAB + M	
	WS‐T0	WS‐T64	WSL‐T0	WSL‐T64	WSLM‐T0	WSLM‐T64
Ash (g kg^–1^ DM)	25.8	22.7*	25.6	22.5*	36.8	38.0*
Crude protein (g kg^–1^ OM)	23.2	24.5	27.6	24.1*	31.9	32.9
Cellulose (g kg^–1^ OM)	494.3	499.3	495.5	500.3	455.4	461.2
Hemicellulose (g kg^–1^ OM)	325.0	298.0*	320.0	311.9	291.7	297.5
Lignin (g kg^–1^ OM)	81.4	74.9*	80.2	74.8*	72.7	71.9
IVGP (mL g^–1^ OM)	192.4	184.8	182.8	177.9	194.2	174.9
Acetic acid (g kg^–1^ DM)	5.70	13.56*	5.41	12.78	6.59	8.35*
NH_3_‐N (g kg^–1^ total N)	61.2	163.6	48.6	263.2*	64.0	95.5*
NaOH amount (mmol kg^–1^ DM)[Link]	33.3	213.5*	34.7	204.9*	55.9	518.9*

* Significantly different (*P* < 0.05) from 64 days to the corresponding value at day 0.

Amount of 0.1 mol L^–1^ NaOH required to increase the pH to 7.

**Table 4:** Chemical composition, in vitro gas production (IVGP) and silage characteristics of autoclaved wheat straw (AWS) stored anaerobically ‘as is’, with the addition of lactic acid bacteria (LAB) or with the addition of a combination of LAB and molasses (LAB + M) for 0 (T0) and 64 (T64) days

Treatment	AWS ‘as is’		AWS + LAB		AWS + LAB + M	
	AWS‐T0	AWS‐T64	AWSL‐T0	AWSL‐T64	AWSLM‐T0	AWSLM‐T64
Ash (g kg^–1^ DM)	24.3	25.0*	24.1	25.0*	35.6	37.7
Crude protein (g kg^–1^ OM)	27.9	28.5	27.8	27.3	31.5	35.9
Cellulose (g kg^–1^ OM)	493.7	491.5	495.4	493.2	447.2	471.8
Hemicellulose (g kg^–1^ OM)	311.9	326.4*	306.8	322.8	284.9	272.7
Lignin (g kg^–1^ OM)	99.1	82.3*	94.3	87.9*	83.8	80.4
IVGP (mL g^–1^ OM)	200.0	192.2	199.6	186.3	212.1	187.0
Acetic acid (g kg^–1^ DM)	6.47	6.33	6.16	7.58*	5.66	7.63*
NH_3_‐N (g kg^–1^ total N)	85.6	88.6	84.9	86.7	82.1	50.3
NaOH amount (mmol kg^–1^ DM)[Link]	41.3	56.0*	41.4	72.7*	55.9	379.5*

* Significantly different (*P* < 0.05) from 64 days to the corresponding value at day 0.

Amount of 0.1 mol L^–1^ NaOH required to increase the pH to 7.

**Table 5:** Chemical composition, in vitro gas production (IVGP) and silage characteristics of C. subvermispora treated autoclaved wheat straw (CS, solid state fermentation for 39 days) stored anaerobically ‘as is’, with the addition of lactic acid bacteria (LAB) or with the addition of a combination of LAB and molasses (LAB + M) for 0 (T0) and 64 (T64) days

Treatment	CS ‘as is’		CS + LAB		CS + LAB + M	
	CS‐T0	CS‐T64	CSL‐T0	CSL‐T64	CSLM‐T0	CSLM‐T64
Ash (g kg^–1^ DM)	24.3	25.2*	24.9	24.9	35.8	36.2
Crude protein (g kg^–1^ OM)	36.3	37.2	35.3	37.8	39.9	41.4
Cellulose (g kg^–1^ OM)	475.2	473.8	475.9	472.4	435.2	427.3
Hemicellulose (g kg^–1^ OM)	165.7	173.4	184.2	179.7	161.3	168.3
Lignin (g kg^–1^ OM)	40.9	35.9*	29.5	34.8	28.6	30.1
IVGP (mL g^–1^ OM)	269.6	268.5	264.5	253.9	263.1	273.5
Acetic acid (g kg^–1^ DM)	1.02	4.93*	0.59	5.35*	0.67	3.45*
NH_3_‐N (g kg^–1^ total N)	1.80	23.6*	1.72	23.6*	10.5	32.3*
NaOH amount (mmol kg^–1^ DM)[Link]	157.3	253.1*	156.0	251.4*	175.4	264.2*

* Significantly different (*P* < 0.05) from 64 days to the corresponding value at day 0.

Amount of 0.1 mol L^–1^ NaOH required to increase the pH to 7.

**Table 6:** Chemical composition, in vitro gas production (IVGP) and silage characteristics of L. edodes treated autoclaved wheat straw (LE, solid state fermentation for 52 days) stored anaerobically ‘as is’, with the addition of lactic acid bacteria (LAB) or with the addition of a combination of LAB and molasses (LAB + M) for 0 (T0) and 64 (T64) days

Treatment	LE ‘as is’		LE + LAB		LE + LAB + M	
	LE‐T0	LE‐T64	LEL‐T0	LEL‐T64	LELM‐T0	LELM‐T64
Ash (g kg^–1^ DM)	25.3	25.3	25.4	25.7	37.7	38.5*
Crude protein (g kg^–1^ OM)	38.7	39.3	41.4	42.0	46.9	47.0
Cellulose (g kg^–1^ OM)	510.0	498.1*	507.5	499.3	456.7	443.8*
Hemicellulose (g kg^–1^ OM)	181.4	182.3	178.9	166.6	166.8	145.1*
Lignin (g kg^–1^ OM)	42.5	42.3	38.0	40.8	33.6	33.6
IVGP (mL g^–1^ OM)	267.6	237.6	267.1	245.7*	251.4	241.9
Acetic acid (g kg^–1^ DM)	1.71	3.18	0.92	3.15*	1.36	4.32*
NH_3_‐N (g kg^–1^ total N)	5.06	25.7*	4.32	24.5*	7.94	31.9*
NaOH amount (mmol kg^–1^ DM)[Link]	157.5	222.7*	161.7	235.5*	175.0	442.9*

* Significantly different (*P* < 0.05) from 64 days to the corresponding value at day 0.

Amount of 0.1 mol L^–1^ NaOH required to increase the pH to 7.

The total IVGP did also not change significantly during anaerobic storage. Only in the case of AWS treated with *L. edodes* and with added *L. plantarum* was total gas production significantly lower by 8.0% after 64 days of preservation compared to 0 days of preservation. Although, for AWS treated with *L. edodes*, a 11.2% decrease was observed. These data show that *C. subvermispora* treated WS can retain its fermentation characteristics as a result of the unchanged fibre composition and its availability for the rumen microbiota. Small changes were observed in IVGP of *L. edodes* treated WS and changes in fibre composition with storage, indicating some degradation. Therefore, from the perspective of chemical composition and gas production, *C. subvermispora* and *L. edodes* treated WS are well preserved anaerobically, either with or without adding LAB and molasses.

#### Concentration of VFA and NH_3_‐N and amount of NaOH required

After the storage period of 64 days, acetic acid was detected in all samples of WS, AWS and fungal‐treated WS (Tables 3–6). Only minor amounts of butyric acid, propionic acid, isobutyric acid, isovaleric acid and valeric acid were detected after 64 days of preservation (data not shown). The treatment with both *C. subvermispora* (Table 5) and *L. edodes* (Table 6) decreased the concentration of acetic acid compared to untreated WS, although the concentration recovered to some extent during the storage period. The same pattern was seen for the NH_3_‐N concentration. During the ensiling of grass and maize, acetic acid and NH_3_‐N are produced by the activity of microorganisms. Although the low pH generated by the fungi will inhibit most microbial growth, it is possible that some microorganisms can still grow and cause changes in NH_3_‐N and acetic acid concentrations.

The buffer capacity of some substrates, such as dried WS, was relatively low, which means that minor amounts of acid could cause a rapid decrease in pH. Therefore, whether the acids produced by the fungi are sufficient for the preservation of the fungal‐treated WS or not is unknown. If the preservation of WS by fungi is mainly caused by the decrease in pH, it is important to estimate the amount and strength of the acids formed. The latter can be achieved by measuring the amount of alkali (NaOH) required to increase the pH to 7.0. For neutralizing the fungal‐treated straw (*C. subvermispora*: 157.3 mmol kg^–1^ DM, *L. edodes*: 157.5 mmol kg^–1^ DM), approximately four times the amount of NaOH was needed compared to the untreated AWS (41.3 mmol kg^–1^ DM). During the 64 days of anaerobic storage, however, the amount of NaOH needed for neutralisation also increased. This correlates with the increase in acetic acid during the storage period. We assume white‐rot fungi to be metabolically inactive during the anaerobic storage period, hence, the increase in NaOH consumption must have another, previously unknown reason. Although the amount of acids increased during storage, this was not detected by a change in pH. The latter can be explained by the fact that pH is expressed on a logarithmic scale and small changes in pH can lead to a significant increase in the alkali required for neutralisation.

## CONCLUSIONS

Both *C. subvermispora* and *L. edodes* improved the fermentability of wheat straw over an 8‐week period as determined by the IVGP. Both fungi substantially decreased the pH during solid state fermentation. The pH decrease <4.3 by *C. subvermispora* and *L. edodes* appears to be effective for stabilising wheat straw, as indicated by the unchanged fibre composition and retained increased fermentability measured with the IVGP method. Fungal‐treated wheat straw can be conserved under anaerobic conditions, without adding lactic acid bacteria and molasses. The latter is highly desirable with respect to the practical use of this technology as a feed resource for ruminants.
